# The self and a close-other: differences between processing of faces and newly acquired information

**DOI:** 10.1093/cercor/bhac201

**Published:** 2022-05-21

**Authors:** Anna Żochowska, Paweł Jakuszyk, Maria M Nowicka, Anna Nowicka

**Affiliations:** Laboratory of Language Neurobiology, Nencki Institute of Experimental Biology, Polish Academy of Sciences, 02-093 Warsaw, Poland; Laboratory of Brain Imaging, Nencki Institute of Experimental Biology, Polish Academy of Sciences, 02-093 Warsaw, Poland; Laboratory of Language Neurobiology, Nencki Institute of Experimental Biology, Polish Academy of Sciences, 02-093 Warsaw, Poland; Laboratory of Language Neurobiology, Nencki Institute of Experimental Biology, Polish Academy of Sciences, 02-093 Warsaw, Poland

**Keywords:** self-preference, attention, saliency, familiarity, ERP

## Abstract

Prioritization of self-related information (e.g. self-face) may be driven by its extreme familiarity. Nevertheless, the findings of numerous behavioral studies reported a self-preference for initially unfamiliar information, arbitrarily associated with the self. In the current study, we investigated the neural underpinnings of extremely familiar stimuli (self-face, close-other’s face) and stimuli newly assigned to one’s own person and to a close-other (abstract shapes). Control conditions consisted of unknown faces and unknown abstract shapes. Reaction times (RTs) to the self-face were shorter than to close-other’s and unknown faces, whereas no RTs differences were observed for shapes. P3 amplitude to the self-face was larger than to close-other’s and unknown faces. Nonparametric cluster-based permutation tests showed significant clusters for the self-face vs. other (close-other’s, unknown) faces. However, in the case of shapes P3 amplitudes to the self-assigned shape and to the shape assigned to a close-other were similar, and both were larger than P3 to unknown shapes. No cluster was detected for the self-assigned shape when compared with the shape assigned to the close-other. Thus, our findings revealed preferential attentional processing of the self-face and the similar allocation of attentional resources to shapes assigned to the self and a close-other.

## Introduction

In order to ensure our adaptive functioning in complex social environments, only some pieces of incoming information are selected for further processing. Such selection is often guided by the self-relevance of the information (Sui and Rotshtein 2019). To start with, a classic example of preferential self-processing is the cocktail party effect (Moray 1959). During a noisy party, even when engaged in an immersive conversation, we can easily hear our own name in the otherwise meaningless noise of other people’s conversations. Numerous studies showed prioritized processing not only of one’s own name (Tacikowski and Nowicka 2010; Yang et al. 2013; Nowicka et al. 2016; Nijhof et al. 2018; Doradzińska et al. 2020) but also for one’s own face (Brédart et al. 2006; Ma and Han 2010; Miyakoshi et al. 2010; Tacikowski and Nowicka 2010; Tacikowski et al. 2011; Kotlewska and Nowicka 2015; Bola et al. 2021; Żochowska et al. 2021). Thus, self-relevance facilitates stimulus processing at different levels: items linked with the self are easier to notice, evaluate, and remember when compared to material associated with other people (e.g. Symons and Johnson 1997; Keyes and Brady 2010; Kotlewska and Nowicka 2016; Nowicka et al. 2016).

Importantly, in the cited studies self-related stimuli were represented by highly familiar items like one’s own name or face. The daily exposure to one’s own face and name across the whole lifespan determines their extreme familiarity in comparison with all other faces and names. Hence, it cannot be clearly differentiated whether the observed effects were caused by the self-relevance or familiarity of those stimuli (Butler et al. 2013; Woźniak et al. 2018; Woźniak and Knoblich 2019; Orellana-Corrales et al. 2021).

To control for the confounding effects of familiarity, Sui and colleagues (Sui et al. 2012) introduced an experimental paradigm that arbitrarily assigned new stimuli to the self and other people. In this task, people formed associations between neutral stimuli (equally familiar) and personally significant labels. Specifically, participants were instructed to associate geometric shapes (e.g. a triangle, a circle, and a square) to the self, a friend, and an unknown other. Subsequently, participants were asked to indicate whether a shape-label pair matched the learned assignment. Response times (RTs) were typically significantly faster for congruent combinations of the self-associated shape and label than when responding to any other shape-label combination. A large prioritization effect was observed not only in RTs, but also in accuracy and sensitivity scores for self-shapes when compared to those of a friend or stranger (Sui et al. 2012; Schäfer et al. 2015; Schäfer et al. 2016; Orellana-Corrales et al. 2021). The immediate and substantial advantage for the self- vs. other pair that was originally reported by Sui and colleagues (Sui et al. 2012) has since been replicated in numerous studies (Frings and Wentura 2014; Mattan et al. 2015; Macrae et al. 2017; Yin et al. 2019).

All in all, a brief association of a neutral shape with the self seems to increase the salience of those stimuli and is sufficient to elicit the self-prioritization effect (Schäfer et al. 2015; Schäfer et al. 2016; Wang et al. 2016; Woźniak and Knoblich 2019). Self-prioritization is thought to influence multiple stages of information processing within matching tasks—the allocation of attention, memory (the retrieval of a self-representation), and decision-making processes (Sui and Humphreys 2015; Humphreys and Sui 2016; Liu et al. 2016). However, the most important account of such self-prioritization is that the effects are driven by tuning attention toward self-related information, i.e. self-relevance modulates attentional processing (Sui and Rotshtein 2019).

However, it is worth noting that in trials with matching pairs of self-associated shape and self-label, participants were processing both self-associated arbitrary stimuli and familiar verbal labels with an established meaning. Therefore, the self-advantage may be caused by the high familiarity of the self-label and not by the self-association of the shape. This fact led some studies to test self-prioritization effects in experimental paradigms with new self- vs. other-associated stimuli only. For example, Sui et al. (2009) associated colors to the self vs. a friend first and then presented arrows in the associated colors at the center of the screen, which served as either valid or invalid cues for the subsequent target location. Arrows in the self-associated color were more efficient in guiding attention than arrows in the friend-associated color. In a similar vein, saccades toward targets positioned away from self vs. other-associated shapes were initiated more slowly (Dalmaso et al. 2019). Moreover, the cuing of target locations by newly self-associated stimuli enhanced target detection (Wade and Vickery 2018; but see Siebold et al. 2015). Finally, the self-prioritization effect could be elicited even in a matching task that employed exclusively neutral stimuli (Woźniak and Knoblich 2019). In that study, participants were first asked to associate avatar faces with three identities: self, friend, and stranger. Afterwards, participants were asked to associate unfamiliar abstract symbols with those three identities. Thus, instead of face-familiar label pairs, pairs of avatar faces, and abstract shapes were presented in a perceptual matching task. Nevertheless, a clear self-prioritization was observed, revealing that this effect can be elicited in the absence of any familiar stimuli. In yet another study, self-prioritization was investigated in an adapted perceptual matching task in which participants were instructed to associate arbitrary stimuli pairs (shape and color) with different people, and then immediately carried out a color-shape matching task. The results showed again the standard pattern of the self-prioritization effect, confirming that the effect is not critically dependent on familiar labels (Lee et al.  2021). In line with the later findings, such effect was observed in a modified matching task, in which familiar labels from the standard task were replaced with pseudo-words, i.e. in the absence of any stimuli with established self-associations (Woźniak and Knoblich 2021). However, it was found only if self-associations were presented as task-relevant (Woźniak and Knoblich 2021).

Most studies investigating the self-prioritization of information that was newly assigned to one’s self vs. another person were based on behavioral measures (RT, accuracy, sensitivity scores). In contrast, studies investigating the neural correlates of such information processing are rather rare (Sui et al. 2013; Sui et al. 2015b; Woźniak et al. 2018). One of the first studies in this field used an associative learning procedure (Sui et al. 2013) that instructed participants to assign three neutral shapes with labels for themselves, their best friend, and an unfamiliar other. Functional magnetic resonance imaging (fMRI) data were acquired while participants judged whether the shape-label pairs matched or not. Behaviorally, faster responses and higher accuracy were found for self-assigned pairs. Responses to matching self-pairs were associated with enhanced activity in the ventral medial prefrontal cortex (vmPFC)—a brain region linked to self-representation (Northoff and Bermpohl 2004; Northoff et al. 2006; Denny et al. 2012)—and in the posterior superior temporal sulcus, which is linked to social cognition (Beauchamp 2015). Activations in those two brain regions predicted behavioral self-bias effects.

In yet another fMRI study (Sui et al. 2015b), participants associated shapes with either themselves or a friend. Subsequently, the shapes had to be identified in hierarchical (i.e. global–local) forms. Self-assigned stimuli were associated with increased activation of the left inferior parietal sulcus when the task required participants to select the neutral shape and ignore the self-associated shape (i.e. salient self-distractors had to be rejected). Since a similar increase in activation in the same region was found when participants rejected perceptually salient distractors (Mevorach et al. 2009), it seems that rapidly formed self-associations may change the neural response in a manner that is qualitatively similar to effects produced by changing the perceptual saliency of stimuli (Sui et al. 2015b).

Further, in an event-related potential (ERP) study three unfamiliar faces were identified with the verbal labels “You,” “Friend,” and “Stranger” instead of shapes (Woźniak et al. 2018). Afterwards, participants judged whether two stimuli (i.e. face, label) presented in succession matched. In one experiment faces were followed by verbal labels, whereas in the other experiment, labels were followed by faces. Both experiments showed an analogous pattern of behavioral and ERP results. If the first stimulus (face or label) was self-related, RTs were faster and the late frontal positivity to the first stimulus was more pronounced. Moreover, the central-parietal P3 associated with the second stimulus was more pronounced when it was preceded by any self-related stimulus. However, when the first stimulus was not associated with the self, there was no facilitation in the processing of the second stimulus even if it had an intrinsic association with the self (Woźniak et al. 2018). Thus, two primary conclusions can be drawn: (1) the self-relevance of initially encountered information has a decisive role in the processing of subsequent information, and (2) self-associated stimuli facilitated the processing of subsequent stimuli, irrespective of whether these stimuli were associated with the self.

In the current ERP study, we investigated the neural underpinnings of highly familiar and new information that was arbitrarily assigned to the self and to a close-other. We were interested in whether previously irrelevant, abstract information that was newly associated with the self would benefit from preferential processing as is the case for well-known self-referential information, and whether self-prioritization effects would be comparable in both cases. Therefore, we directly compared the processing of two types of stimuli: extremely familiar (self-face, close-other’s face) stimuli, and stimuli that were newly assigned to one’s own person and to a close-other (an abstract shape). The control conditions consisted of unknown faces and unknown abstract shapes. We decided to present participants with abstract shapes alone, without labels. The reason for doing so was twofold. First, this approach avoids the aforementioned controversies regarding familiar labels. Second, as there was no need to use any labels in the case of faces, this approach (i.e. avoiding labels) guaranteed similar visual stimulation in both cases.

Participants were tasked with indicating whether presented stimuli (faces, shapes) were familiar or unknown. Prior to the EEG recording session, arbitrarily selected shapes were associated with the self and a close-other (i.e. one shape for each person). The close-other was freely chosen by each participant as representative of the most significant person in their life at the time of experimentation. This operationalization of a close-other was used in several previous studies on the topic of self-prioritization (Cygan et al. 2014; Kotlewska and Nowicka 2015; Kotlewska and Nowicka 2016; Nowicka et al. 2016; Kotlewska et al. 2017; Nijhof et al. 2018; Nowicka et al. 2018; Cygan et al. 2021). It is worth noting that similarly to one’s own face, a close-other’s face is a very important and salient visual stimulus that is frequently encountered on an everyday basis. Thus, its level of familiarity is very high. Nevertheless, on the neural level the processing of such extremely familiar faces—with a very high exposure factor—substantially differs from the processing of the self-face as revealed by steady-state visual evoked potentials (Kotlewska et al. 2017) and late ERP components, especially P3—a positive ERP component with centro-parietal distribution and latency of 300 ms or longer (Cygan et al. 2014; Kotlewska and Nowicka 2015; Cygan et al. 2021).

In this study, the P3 results obtained for faces served as a kind of reference for ERP results for shapes. We expected to observe enhanced P3 associated with self-face processing when compared to close-other and unknown face processing. We aimed at testing whether information newly assigned to the self and a close-other would lead to an analogous pattern of findings. As far as behavioral indices of self-prioritization are also concerned, we were interested in whether behavioral findings would be comparable for faces and shapes.

Moreover, the distinct spatial patterns of activity elicited by faces and shapes were also tested with nonparametric cluster-based permutation tests (Maris and Oostenveld 2007). This method enables the unbiased comparison of EEG signals recorded in different experimental conditions at all sensors and all time points, while controlling for multiple comparisons and maximizing power by employing the cluster structure of the data as its sole test statistic. We used this approach to test for differences in spatial and temporal distributions between experimental conditions. Thus, cluster-based permutation tests would confirm and complement the findings obtained with P3 analyses, providing a global and complete view of commonality/distinctiveness in the neural underpinnings associated with the processing of self-, close-other, and unknown faces and newly acquired information referring to the self, a close-other, and unknown people.

## Materials and methods

### Participants

Thirty-two participants (16 females, 16 males) were tested in the study, ranging in age between 21 and 34 years old (*M* = 27.594; SD = 3.131). The Edinburgh Handedness Inventory (Oldfield 1971) indicated that thirty participants were right-handed. All participants reported no history of mental or neurological disorders and had normal or corrected-to-normal vision with the use of contact lenses. Additionally, to ensure the uniformity of visual stimuli standards, neither participants nor their chosen close-other were allowed to be represented with glasses or have any distinctive facial marks, as their photographs were matched with photographs from the Chicago Face Database (CFD; Ma et al. 2015).

An additional present-day restriction was a negative test for the SARS-CoV-2 virus. As all participants were PhD students and employees at the Nencki Institute, they took part in the SONAR-II project (www.nencki.edu.pl) developed at the Nencki Institute in cooperation with the University of Warsaw. SONAR-II is dedicated to the asymptomatic population of people who do not meet the criteria for SARS-CoV-2 testing but who may come into contact with infected people.

The required sample size was estimated using MorePower software (Campbell and Thompson 2002). Estimation was conducted for the main factor “type of stimuli” (face, shape) in two-way repeated measures ANOVA with the factors “type of stimuli” and “type of face” (self, close-other’s, unknown): estimated effect size η^2^ = 0.25, α = 0.05, β = 0.90. It yielded a sample size of 30 participants. As the risk of data loss was taken into consideration, the group size was enlarged to 32.

### Ethics statement

This study was conducted with the approval of the Human Ethics Committee of the Institute of Applied Psychology at Jagiellonian University (Cracow, Poland). All participants provided written informed consent prior to the study and received financial compensation for their participation.

### Stimuli

We used two different types of stimuli in this study: (1) faces and (2) shapes. The set of stimuli was individually tailored to each participant. Faces belonged to three categories: self-face, close-other’s face, and unknown faces. Participants freely chose the close-other according to their subjective high level of closeness and importance. We did not predefine the type of relationship between the participant and their close-other in order to avoid a spuriously close relation. The only restriction placed on the selection of the close-other was that they had to be of the same gender as the participant. Twenty-two participants chose their friend, eight their sibling, and two their partner. The face of each participant and their close-other was photographed (with a neutral expression) prior to the study. The photographs of eight unknown neutral faces were taken from the Chicago Face Database (Ma et al. 2015), gender matched to each participant. Each photo (the self-face, close-other’s face, and selected unknown faces from the CFD) was subjected to the same editing procedure, i.e. they were gray-scaled, extracted from the background and cropped (only facial features were included—face oval without hair and ears), resized to subtend 6.7° × 9.1° of visual angle, and equalized for mean luminance using Photoshop CS5 (Adobe, San Jose, CA). Contrast and spatial frequencies in the pictures were not normalized as these procedures tend to introduce substantial distortions into the processed images. The photos of each participant and their close-other’s face were deleted at the end of the experimental session.

**Fig. 1:**
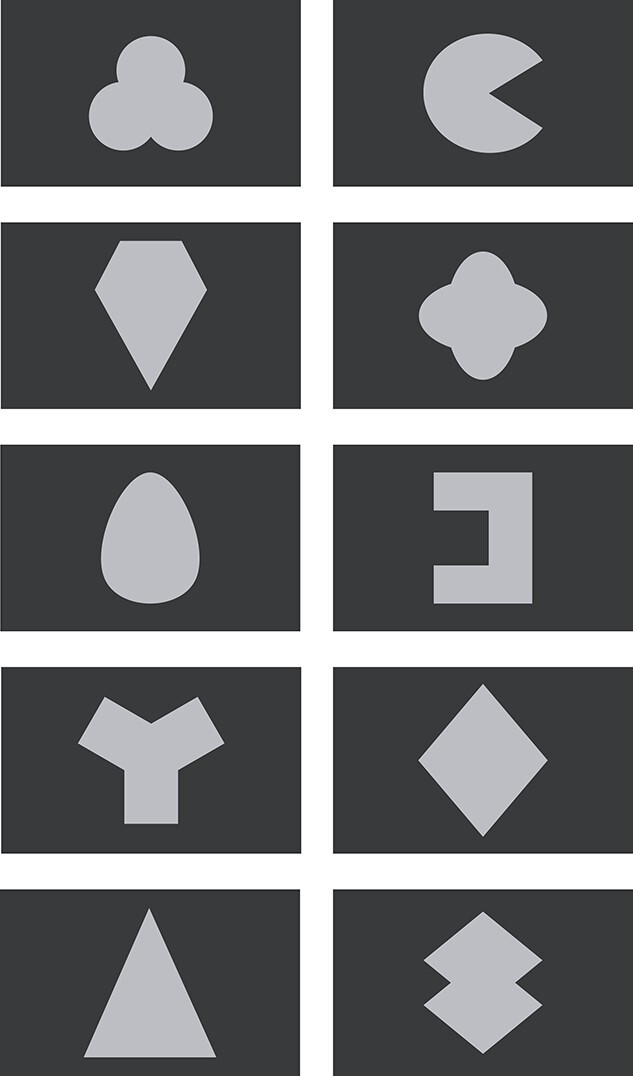
Shapes used in the present study. The area of each shape was the same and was equal to the area of the face oval.

The second type of stimuli consisted of abstract shapes. In previous studies on the processing of new information assigned to the self and others, simple geometric figures (e.g. a square, a triangle) were typically used. As the number of shapes was supposed to be equal to the total number of faces (self, close-other’s, 8 unknown) we generated 10 different abstract shapes. We aimed at equalizing low-level physical features of faces and shapes. Thus, each shape’s area was equal to face oval’s area, i.e. 43.12 cm^2^. Shape assignment to each experimental “condition” (self, close-other, unknown) was pseudo-random on the group level; e.g. a self-assigned shape in a given set of stimuli was assigned to a close-other or unknown condition in some other set of stimuli. Faces and shapes were presented against a black background. Figure 1 presents all 10 shapes used in this study.

### Procedure

Participants were seated in a comfortable chair in a dimly lit and sound-attenuated room, 57 cm from the computer monitor (DELL Alienware AW2521HFL, Round Rock, Texas, USA). Subsequently, during the electrode cap placement and adjustment of EEG electrode impedances, they were primed for the task: two different shapes, one assigned to the participant and the second to their close-other, were presented on the monitor and participants were required to associate those geometric shapes with the self and chosen close-other. The learning phase lasted on average 23.53 min (SD = 5.900). Just before the beginning of the task, participants were asked to draw the assigned shapes. This was done to check the efficiency of learning.

After electrode cap placement (ActiCAP, Brain Products, Munich, Germany), the participants used an adjustable chinrest to maintain a stable head position. Presentation software (Version 18.2, Neurobehavioral Systems, Albany, CA) was used for stimuli presentation. Participants performed a recognition task—if they recognized a presented face or shape (i.e. representing the participant or their close-other), they were asked to push the response button assigned to “YES.” If that was not the case, they were asked to press the button defined as “NO.” The assignment of “YES” and “NO” buttons was counterbalanced across the participants.

**Fig. 2:**
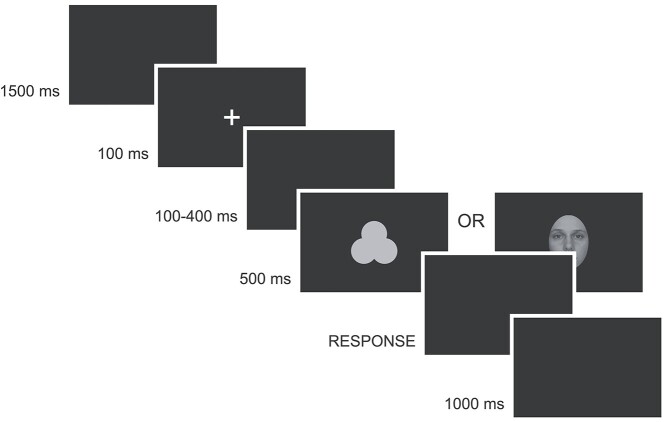
Schematic presentation of the experimental procedure. Three types of faces (self, close-other’s, unknown) and three types of shapes (self-assigned, assigned to the close-other, unknown) were intermixed and presented pseudo-randomly. Participants were supposed to indicate whether a stimulus was familiar or not. The example image of a self-face is a photograph of one of the co-authors.

After reading the instructions displayed on the screen, the subjects confirmed they understood the task and initiated the experiment by pressing a response button. Trials with faces and shapes were inter-mixed (in one session) and their order was pseudo-random with respect to the type of stimulus (faces, shape) and the experimental condition (self, close-other, unknown). Each trial started with a blank screen, presented for 1500 ms. Next, a white fixation cross (subtending 0.5° × 0.5° of visual angle) was centrally displayed for 100 ms and followed by a blank screen which lasted either (randomly) 100, 200, 300, or 400 ms. Afterwards, a face or a shape was presented for 500 ms. Regardless of which stimulus was shown, participants were asked to push the appropriate response button as quickly as possible. Next, a blank screen was shown and lasted until a response was made. The procedure structure is presented in Fig. 2. In the “self” and “close-other” conditions, the total number of repetitions for each stimulus type (face, shape) was 40, while for the “unknown” condition it was 80. Thus, the total number of trials with familiar and unknown stimuli was equal, as was the probability of YES/NO responses.

To account for possible fatigue during the experiment, a break was planned in the middle of experimental session. It was terminated after one minute, unless the participant ended it earlier. Participants needed 24 min on average to complete the whole experiment.

### E‌EG recording

The electroencephalogram (EEG) was continuously recorded with 62 Ag-AgCl electrically shielded electrodes mounted on an elastic cap (ActiCAP, Brain Products, Munich, Germany) and positioned according to the extended 10–20 system, with two additional electrodes placed on the left and right earlobes. The EEG signal was recorded using the BrainAmp MR plus amplifier (Brain Products, Munich, Germany) and digitized at a 500-Hz sampling rate, using BrainVision Recorder software (Brain Products, Munich, Germany). EEG electrode impedances were kept below 10 kΩ. The EEG signal was recorded against an average of all channels calculated by the amplifier hardware.

### Behavioral data analysis

Responses were scored as correct if the appropriate button was pressed within 100–1000 ms of stimulus onset. In order to conduct statistical analyses of behavioral and ERP data in a consistent manner, similarly to our analyses of ERP components, only every other trial with an unfamiliar stimulus was included in the analyses. This was done because the number of trials with unfamiliar faces (80) and shapes (80) was doubled in comparison to the number of trials with familiar faces and shapes in each familiar (self, close-other) condition (40). Statistical analyses were performed using JASP software (Wagenmakers et al. 2018). Mean accuracy scores and mean RTs were analyzed using repeated measures ANOVA, with the “type of stimulus” (face, shape) and “condition” (self, close-other, unfamiliar) as within-subject factors.

The traditional null-hypothesis significance-testing approach was complemented with Bayesian analysis methods and Bayes factors (BFs) were computed using JASP software (Wagenmakers et al. 2018). BFs were interpreted according to Lee and Wagenmakers (2014) suggestions. Briefly, a BF_10_ between 1 and 3 implies anecdotal evidence in favor of H1, between 3 and 10—moderate evidence, between 10 and 30—strong evidence, between 30 and 100—very strong, and higher than 100—extreme evidence. As far as low values of BF_10_ are concerned, a BF_10_ between 0.33 and 1 indicates anecdotal evidence in favor of H0, between 0.1 and 0.33—moderate evidence, and between 0.03 and 0.1—strong evidence of the absence of an effect. Finally, a BF_10_ between 0.01 and 0.03 and lower than 0.01 indicates very strong and extreme evidence for the absence of an effect, respectively.

### ERP analysis

Off-line analysis of the EEG was performed using BrainVisionAnalyzer software (Brain Products, Gilching, Germany). The 62 channels were re-referenced off-line to the algebraic average of the left and right earlobes, notch filtered at 50 Hz, and band-pass- filtered from 0.1 to 30 Hz using a Butterworth filter. The next step in data pre-processing was the correction of ocular artifacts using Independent Component Analysis (ICA) (Bell and Sejnowski 1995). After each data set was decomposed into maximally statistically independent components, elements representing eye blinks were rejected based on a visual inspection of the component’s map (Jung et al. 2001). Using the reduced component-mixing matrix, the remaining ICA components were multiplied and back-projected to the data, resulting in a set of ocular-artifact-free EEG data.

Afterward, the EEG signal was segmented into 1200 ms epochs, from −200 ms before to 1000 ms after stimulus onset. The subsequent automatic artifact rejection procedure allowed only trials, which fulfilled the following requirements: the maximum permitted voltage step per sampling point was 50 μV, the maximum permitted absolute difference between two values in the 200-ms-long segment was 100 μV, the minimal and maximal allowed amplitudes were −150 μV and 150 μV, and the lowest permitted activity in the 100 ms interval was 0.5 μV.

Trials with correct responses were subsequently analyzed. In the case of unfamiliar stimuli, only every other trial was included in the analyses. This was done in order to keep a similar signal-to-noise ratio (SNR) for each experimental condition (defined by the type of stimulus and type of face). It should be reminded that the total number of trials with unfamiliar faces and shapes (80 for each of them) was twice as large as the total number of trials with familiar faces and shapes in the single “self” and “close-other” conditions (40 for each type of stimulus). The mean number of segments averaged afterwards for each experimental condition was as follows: self-face—37.031 (SD = 2.800), shape assigned to self—35.250 (SD = 3.298), close-other’s face—36.938 (SD = 2.449), shape assigned to close-other −35.031 (SD = 3.441), unknown other face—36.344 (SD = 2.868), and unknown shape—35.938 (SD = 3.816). In the final stage of pre-processing, the epochs were baseline-corrected by subtracting the mean of the pre-stimulus period.

**Fig. 3:**
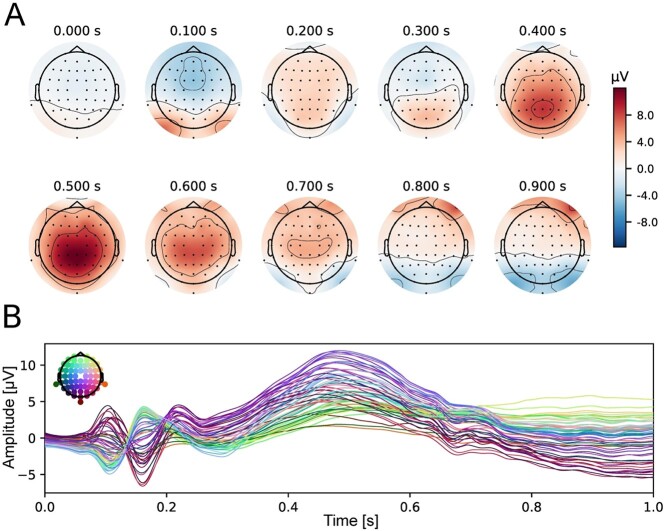
(A) Topographical distribution of brain activity averaged across the two types of stimuli (faces, shape) and across all experimental conditions (self, close-other, unknown) and (B) a butterfly plot presenting grand-average ERPs for all (collapsed) experimental conditions, at all 62 active electrodes.

Selection of electrodes for ERP analysis was orthogonal to potential differences between experimental conditions (Kriegeskorte et al. 2009). Therefore, it was conducted on the basis of the topographical distribution of brain activity (in the time window corresponding to a given component), averaged across all experimental conditions. Electrodes CP1, CPz, CP2, and Pz, localized within the region of maximal activity, were selected for further analyses (see Fig. 3). The data were pooled for those electrodes. This step is justified by the limited spatial resolution of EEG and high correlation between neighboring electrodes. Based on the topographical distribution of activity as well as grand-averaged ERPs, collapsed for all experimental conditions (self-face, shape assigned to the self, close other’s face, shape assigned to close other, unknown other face, and unknown shape), a 350–650-ms time window was chosen for analysis of the P3 component. The mean values at each time point within this time window were used to assess the amplitudes of our ERP component. This method is less affected by possible low SNR than peak measure methods (Luck 2005).

Statistical analysis of ERP data was performed using SPSS software (Version 26, IBM Corporation). The reported results were cross-checked with Statcheck (http://statcheck.io/index.php). A two-way repeated measure ANOVA was performed with “type of stimulus” (face, shape) and “condition” (self, close-other’s, unknown) as within-subject factors. All effects with more than one degree of freedom in the numerator were adjusted for violations of sphericity (Greenhouse and Geisser 1959). Bonferroni correction for multiple comparisons was applied to post hoc analyses. All results are reported with alpha levels equal to 0.05.

The traditional null hypothesis significance testing approach was complemented with Bayesian analysis methods. To test whether the self-face and other faces, as well as the self-assigned shape and other shapes, were characterized by similar levels of neural activity, BFs were computed using JASP software (Wagenmakers et al. 2018). The main reason for calculating BFs was that, unlike classic frequentist statistics, BF evaluates how strongly both alternative and null hypotheses are supported by the data. Specifically, BF is a ratio of the probability (or likelihood) of observing the data given the alternative hypothesis is true to the probability of observing the data given the null hypothesis is true. Thus, BF_10_ provides further evidence either in favor of similarities or rather differences between the tested experimental conditions. The medium prior scale (Cauchy scale 0.707) was used in all Bayesian tests. BF_10_ were interpreted according to Lee and Wagenmakers (2014) suggestions.

### Cluster-based permutation tests

Cluster-based permutation tests were used here as an exploratory analysis procedure, as they efficiently handle the multiple comparisons problem in high-dimensional magnetoencephalographic and EEG data (Sassenhagen  and Draschkow 2019). In general, permutation tests are used to test the null hypothesis that the data in the experimental conditions come from the same probability distribution. Getting a significant result means that the null hypothesis can be rejected in favor of the alternative hypothesis, i.e. that the data came from different distributions. Therefore, significant results from permutations tests indicate a significant between-condition difference. The results are reported with reference to an alpha level equal to 0.05. Cluster-based permutation tests were conducted using custom-made Python scripts with use of the mne.stats.spatio_temporal_cluster_1samp_test function from the MNE Python package.

We directly compared: self-face vs. close-other’s face, self-face vs. unknown faces, close-other’s face vs. unknown faces, self-shape vs. close-other’s shape, self-shape vs. unknown shapes and close-other’s shape vs. unknown shapes. As clustering in both space and time was used, such an analysis procedure revealed differences in the spatial distributions of activity as a function of time between the tested conditions.

## Results

### Behavioral results

**Fig. 4:**
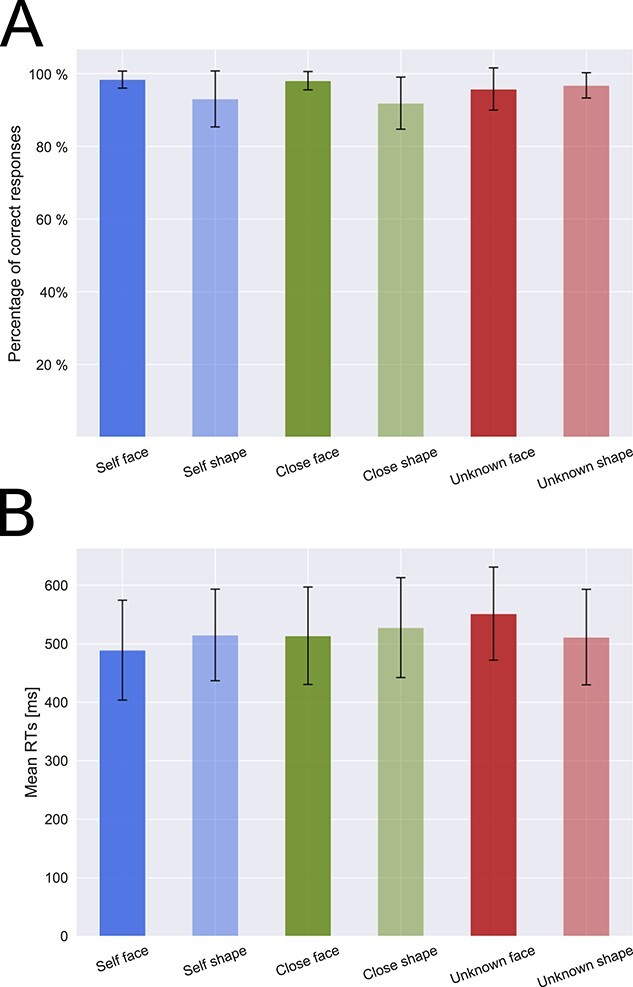
Behavioral results. (A) Mean (± *SD*) accuracy scores and (B) mean (± *SD*) RTs for faces and shapes.

A repeated-measures ANOVA conducted on the mean number of correct responses revealed the significant main effects of “type of stimulus” (*F*(1, 31) = 28.758, *P* < 0.001, η^2^ = 0.141) and “condition” (*F*(2, 62) = 4.022, *P* = 0.023, η^2^ = 0.028), as well as a significant two-way interaction: “condition” × “type of stimulus” (*F*(2, 62) = 10.689, *P* < 0.001, η^2^ = 0.119). The significance of the “type of stimulus” factor indicated a significantly higher accuracy score in the case of faces in comparison with shapes (see Fig. 4). Post hoc tests of the “condition” factor showed that the accuracy score in the “close-other” condition was slightly lower than in the “unknown” condition (*P* = 0.020), whereas other differences were non-significant (“self” vs. “close-other”: *P* = 0.808; “self” vs. “unknown”: *P* = 0.282).

Post-hoc tests of the “condition” × “type of stimulus” interaction revealed non-significant differences in accuracy scores between the self-face and the close-other’s face (*P* > 0.99, BF_10_ = 0.231, moderate evidence for H0), the self-face and unknown faces (*P* = 0.933, BF_10_ = 0.597, anecdotal evidence for H0), and the close-other’s face vs. unknown faces (*P* > 0.999, BF_10_ = 0.541, anecdotal evidence for H0). Significant differences in accuracy rates were present between the self-assigned shape and unknown shapes (*P* = 0.002, BF_10_ = 15.894, strong evidence for H1) and between the close-other assigned shape and unknown shapes (*P* < 0.001, BF_10_ = 210.730, extreme evidence for H1), whereas the self-assigned shape and the close-other assigned shape did not differ (*P* > 0.999, BF_10_ = 0.256, moderate evidence for H0).

Moreover, post hoc tests of the “condition” × “type of stimulus” interaction also showed that the number of correct responses to faces was significantly higher than to shapes in the “self” and “close-other” conditions (*P* < 0.001, BF_10_ = 50.757, very strong evidence for H1 and *P* < 0.001, BF_10_ = 6596.037, extreme evidence for H1, respectively), but it was similar for faces and shapes in the case of the “unknown” condition (*P* > 0.99, BF_10_ = 0.297, moderate evidence for H0).

A repeated-measures ANOVA conducted on mean RTs revealed the significant main effect of “condition” (*F*(2, 62) = 25.374, *P* < 0.001, η^2^ = 0.159) and a significant two-way interaction: “condition” × “type of stimulus” (*F*(2, 62) = 36.036, *P* < 0.001, η^2^ = 0.230). Post hoc tests of the “condition” factor showed that RTs in the “self” condition were substantially shorter than in the “close-other” (*P* < 0.001) and “unknown” conditions (*P* < 0.001). However, this pattern of findings was driven mainly by RTs to faces. Post hoc tests of the “condition” × “type of stimulus” interaction revealed significantly shorter RTs to the self-face than to the close-other’s face (*P* = 0.001, BF_10_ = 4517.073, extreme evidence for H1) and unknown faces (*P* < 0.001, BF_10_ = 1.009 × 10^10^, extreme evidence for H1), as well as shorter RTs to the close-other’s face than to unknown faces (*P* < 0.001, BF_10_ = 5.644 × 10^6^, extreme evidence for H1). In contrast, in the case of shapes, all differences between conditions were non-significant (self vs. close-other: *P* = 0.624, BF_10_ = 0.637, anecdotal evidence for H0; self vs. unknown: *P* > 0.99, BF_10_ = 0.227, anecdotal evidence for H0; close-other vs. unknown: *P* = 0.105, BF_10_ = 2.414, anecdotal evidence for H1).

Post hoc tests of the “condition” × “type of stimulus” interaction also showed that RTs to the self-face were significantly shorter than to the self-assigned shape (*P* = 0.009, BF_10_ = 22.438, strong evidence for H1). The opposite effect, i.e. longer RTs, was observed for unknown faces when compared to unknown shapes (*P* < 0.001, BF_10_ = 1.227 × 10^7^, extreme evidence for H1), but no significant differences were found in the case of the “close-other” condition (*P* = 0.908, BF_10_ = 0.569, anecdotal evidence for H0).

### P3 results

**Fig. 5:**
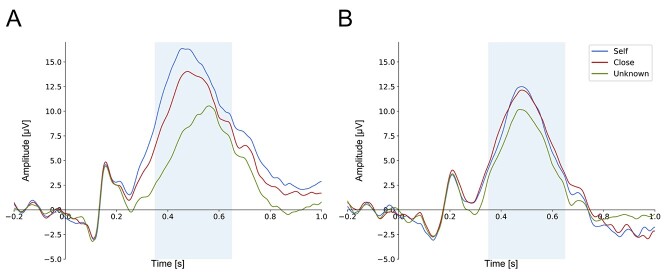
Grand-average ERPs for (A) faces and (B) shapes, pooled for four electrodes: CP1, CPz, CP2, and Pz. The analyzed time window is marked by light-blue rectangles.

Statistical analysis of P3 amplitudes revealed the main effects of “type of stimulus” (*F*(1, 31) = 27.004, *P* < 0.001, η^2^ = 0.466), “condition” (*F*(2, 62) = 32.288, *P* < 0.001, η^2^  = 0.510), and a significant 2-way “condition” × “type of stimulus” interaction (*F*(2, 62) = 15.514, *P* < 0.001. η^2^ = 0.334). The significance of the “type of stimulus” factor indicated that the P300 amplitude for faces was significantly higher than for shapes (see Fig. 5).

Post-hoc analyses for the “condition” factor showed that the unknown stimuli evoked significantly lower P3 than stimuli associated with the self (*P* < 0.001) as well as for stimuli associated with the close-other (*P* < 0.001). The difference between stimuli associated with self and close-other was non-significant (*P* = 0.221).

Post hoc tests performed for the two-way “condition” × “type of stimulus” interaction revealed that the self-face was associated with significantly higher P3 than the shape assigned to the self (*P* < 0.001, BF_10_ = 247366.068, extreme evidence for H1). This was also the case for the close-other condition: P3 to the close-other’s face was larger than P3 to the shape assigned to a close-other (*P* < 0.001, BF_10_ = 84.268, very strong evidence for H1). Such an effect was not observed for unknown stimuli, as the difference between unknown faces and unknown shapes was not significant (*P* = 0.165, BF_10_ = 0.470, anecdotal evidence for H0). Moreover, P3 amplitude was significantly increased for self-face in comparison to close-other’s face (*P* = 0.008, BF_10_ = 13.409, strong evidence for H1) as well as in comparison to unknown faces (*P* < 0.001, BF_10_ = 4.871 × 10^6^, extreme evidence for H1), and for close-other’s face compared to unknown faces (*P* = 0.001, BF_10_ = 7478.356, extreme evidence for H1). P3 amplitudes to the self-assigned shape and the close-other assigned shape did not differ (*P* > 0.999, BF_10_ = 0.207, moderate evidence for H0). However, unknown shapes were associated with lower P3 amplitude than the shape assigned to the self (*P* = 0.009, BF_10_ = 12.049, strong evidence for H1) and to the close-other (*P* = 0.004, BF_10_ = 25.077, strong evidence for H1).

### Cluster-based permutation tests

**Fig. 6:**
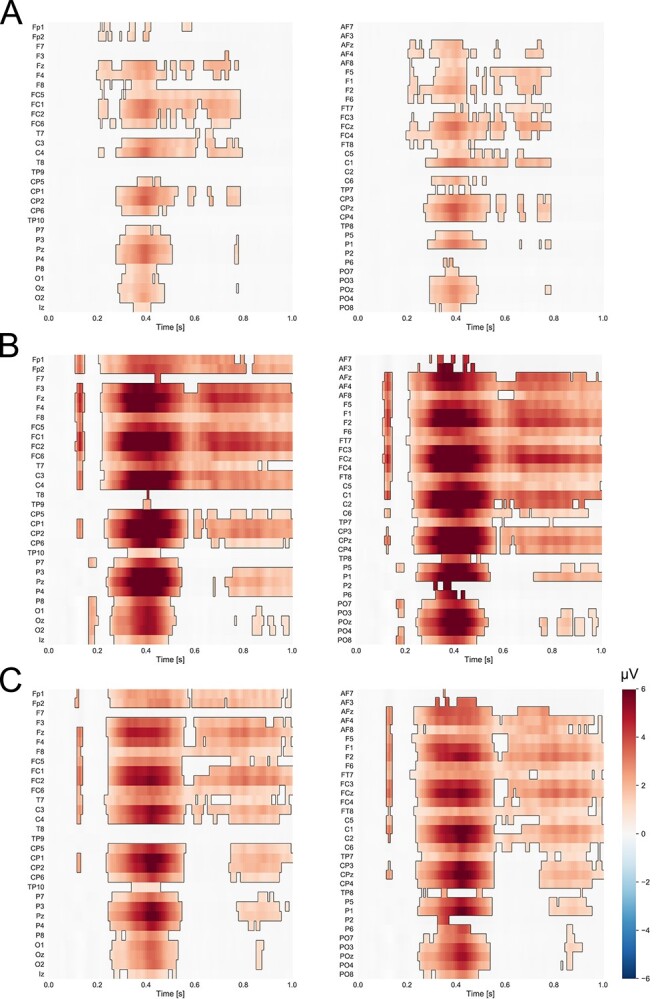
The results of cluster-based permutation tests for faces. (A) Self-face compared to close-other and (B) unknown faces, (C) close-other face compared to unknown faces. Statistically significant positive differences between conditions are indicated in red (*P* < 0.05).

Nonparametric cluster-based permutation analyses showed that the self-face processing differed significantly from the processing of all other faces, i.e. close-other’s and unknown faces. Differences between the self- and unknown faces as well as between the close-other’s face and unknown faces were widely distributed in space and time, whereas a significant cluster was more focused for the self vs. the close-other comparison (see Fig. 6). It is worth noting that the time window of substantial differences between the tested conditions encompasses the time window in which the P3 component was analyzed (350–650 ms). Moreover, differences between conditions were present at electrodes within the central-parietal region for all comparisons, i.e. the region for which P3 amplitudes were analyzed.

**Fig. 7:**
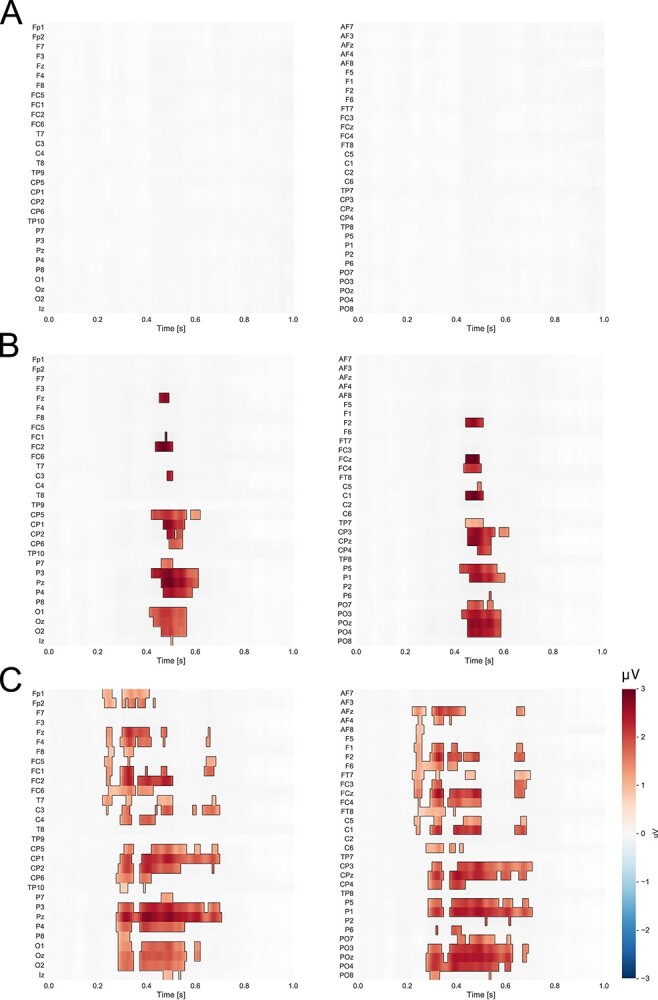
The results of cluster-based permutation tests for shapes. The self-assigned shape compared to the shape assigned to (A) the close-other and (B) unknown shapes, and (C) the shape assigned to the close-other compared to unknown shapes. Statistically significant positive differences between conditions are indicated in red (*P* < 0.05).

In the case of abstract shapes, nonparametric cluster-based permutation analyses revealed significant differences between the self-assigned shape vs. unknown shapes and the close-other assigned shape vs. unknown shapes (see Fig. 7). Crucially, when compared to the shape assigned to the close-other, no difference was detected in the case of the self-assigned shape, at any electrode site and at any time point (see Fig. 7). Such a lack of the differences indicates that the data in those two experimental conditions (self, close-other) came from the same probability distribution (i.e. the data in these conditions cannot be distinguished).

## Discussion

Converging lines of evidence indicate that self-relevance facilitates stimulus processing and different types of self-related information (e.g. name, face) are processed preferentially (for a review see Humphreys and Sui 2016). There is an ongoing debate on whether such a self-advantage can be attributed to the extreme familiarity of self-related information and whether the processing advantages for self-related information can be observed for initially unfamiliar information, when arbitrarily associated with the self (Sui et al. 2012).

The current study investigated the neural underpinnings and behavioral indices of the processing of self- and close-other’s faces as well as for abstract shapes that were—prior to the experimental session—assigned to the self and a close-other. It should be pointed out that the close-other condition seems to be the best control to the self and has been used in several previous studies on self-referential processing (Cygan et al. 2014; Kotlewska and Nowicka 2015; Kotlewska and Nowicka 2016; Nowicka et al. 2016; Kotlewska et al. 2017; Nijhof et al. 2018; Nowicka et al. 2018; Cygan et al. 2021). The processing of those two types of faces and shapes was compared with the processing of unknown faces and shapes.

On the behavioral level, we observed a rather complex pattern of findings. To start with, accuracy rates were significantly higher for faces than for shapes in the “self” and “close-other” conditions, but they were similar for faces and shapes in the “unknown” condition. Accuracy scores to the self-assigned shape and the shape assigned to the close-other did not differ. However, both were lower than accuracy score to unknown shapes. Even so, it should be pointed out that accuracy rates were nominally very high in each experimental condition (from 92 to 99%). RTs analyses revealed that all self-related stimuli were characterized by shorter RTs in comparison to the other stimuli (i.e. referring to the close-other and unknown people). Additionally, it should be highlighted that this result was primarily driven by the RTs to faces—significant differences were observed between self-face and other faces, but not between shapes. Moreover, RTs to the self-face were significantly shorter than to the self-assigned shape. Such faster reactions to faces than to shapes were not observed in the case of the “close-other” and “unknown” conditions.

Based on numerous studies showing behavioral indices of the self-prioritization effect for new information that is arbitrarily associated with the self (Sui et al. 2012; Sui et al. 2013; Frings and Wentura 2014; Sui et al. 2014; Mattan et al. 2015; Schäfer et al. 2015; Sui et al. 2015a; Schäfer et al. 2016; Macrae et al. 2017; Woźniak et al. 2018; Yin et al. 2019; Orellana-Corrales et al. 2021), one might also expect the current study to find shorter RTs and higher accuracy rates in the case of newly self-assigned stimuli. However, this was not the case. This discrepancy may be due to the substantial procedural/methodological differences between the previous studies and the current study.

To start with, one of the differences refers to the presence vs. absence of labels. Specifically, in the matching trials of the self-prioritization task used in previous studies, participants were processing not only self-associated arbitrary stimuli but also familiar verbal labels with a pre-experimentally established meaning. Therefore, the self-advantage may be caused by the familiarity of the labels, rather than the self-association of the shape. In contrast, the present study did not present labels with shapes. However, it should be noted that the self-prioritization effect in RTs was also observed in the absence of any pre-experimentally familiar stimuli related to the self (Woźniak and Knoblich 2019). Thus, the RT findings in the current study and in Woźniak and Knoblich (2019) may be regarded as inconsistent. However, the next difference between the current and previous studies on the topic of processing newly acquired information (including Woźniak and Knoblich 2019) is that in our study the control condition to the self was not just a “friend” but a person, freely chosen by each participant as the most significant person at the time of experimentation. Other differences are as follows. In the current study, shapes assigned to the self and the close-other were presented for a rather prolonged time (ca. 30 min), whereas in previous studies the process of associating a specific shape to a specific person was much shorter: each participant was simply told that they would be represented by e.g. a circle or a square (Sui et al. 2012) or the learning phase of shapes labels was very short—30 or 60 s (Woźniak and Knoblich 2019).

Crucially, the behavioral tasks performed by participants were different. While in previous studies, it was the perceptual matching task, in the current study it was the discrimination of familiar vs. unfamiliar stimuli. Thus a question may arise whether participants ignored self- and close-other associations when responding to the shapes and simply re-conceptualized those shapes as simply representing the category “familiar.” Such strategy was fully efficient in successfully accomplishing the task and accuracy rates seemed to support this view as they did not differ for self- and close-other assigned shapes.

It is worth noting that the self-relevance facilitated stimulus processing only when task sets drew attention to previously formed shape-label associations (Caughey et al. 2021). Compared to shapes associated with a friend, those paired with the self were classified more rapidly when participants were required to report who the stimulus denoted (i.e. self or friend). However, self-relevance failed to facilitate performance when participants judged either what the shape was (i.e. triangle or square, diamond or circle) or where it was located on the screen (i.e. above or below fixation). This was also the case for arbitrary objects assigned to the self and a friend (Falben et al. 2019). Compared with arbitrary objects owned by a friend, those owned by the self were classified more rapidly when participants were required to report either the owner or identity of the items. In contrast, self-relevance failed to facilitate performance when participants judged the orientation of the stimuli. In a similar vein, the self-prioritization effect was observed (in the absence of any stimuli with established self-associations) only when self-associations were task-relevant (Woźniak and Knoblich 2021). In the light of the aforementioned findings, behavioral results for shapes assigned to the self and a close-other were similar because the self-association of a shape was task-irrelevant as it was not necessary to identify shapes as associated with the self or a close-other.

Even so, we found faster reactions to one’s own face than to other faces. Our RTs results are in line with the findings reported in numerous studies, typically reporting shorter RTs to the self vs. other faces (Keyes et al. 2010; Ma and Han 2010; Tacikowski and Nowicka 2010; Żochowska et al. 2021). In a recent meta-analysis, RTs to the self-face were compared with RTs to other faces across a large number of studies (Bortolon and Raffard 2018). The tested moderators included—among others—the familiarity (i.e. whether the face was familiar to the participants) and identity of faces (i.e. whether the face belonged to someone personally known by participants, or whether it was a famous person or a stranger). The results of that meta-analysis showed that regardless of the face identity or level of familiarity, people tended to respond faster to their own face than to other people’s faces when requested to perform an identification/recognition task (Bortolon and Raffard 2018).

On the neural level, the ERP findings differed for faces and shapes. First of all, amplitudes of P3 to faces were—in general—higher than amplitudes of P3 to shapes. As the P3 component is linked to the cognitive evaluation of stimulus significance (Picton and Hillyard 1988), this finding may suggest the increased significance of faces in comparison with abstract shapes. While the former are ecologically valid stimuli that are encountered on an everyday basis, the latter definitely do not share those features. In addition, the ERP results of the present study clearly showed that self-face processing was associated with enhanced P3 in comparison with all other faces (close-other’s, unknown). Furthermore, P3 to the close-other’s face was larger than P3 to unknown faces. Nonparametric cluster-based permutation tests corroborated our P3 findings, as they revealed significant clusters for the self-face when compared to both the close-other’s face and unknown faces, as well as for the close-other’s face when compared to unknown faces.

However, the pattern of findings was different in the case of shapes. P3 to the self-assigned shape and P3 to the close-other assigned shape were similar, and both were larger than P3 to unknown shapes. Moreover, nonparametric cluster-based permutation tests showed significant clusters for comparisons of “self vs. ‘unknown’ and ‘close-other” vs. “unknown” conditions, but no significant cluster was detected for the self-assigned shape when compared to the close-other assigned shape. The latter is in line with the lack of differences in P3 amplitude between the self- and close-other conditions.

Due to methodological differences, it is rather difficult to directly compare our P3 findings for the self-assigned shape to previous ERP findings on the processing of newly acquired self-related information (Woźniak et al. 2018). In Woźniak et al.’s study with the matching task of labels and previously unknown faces, associated with the self and others, self-association of the first stimulus in a pair determined the pattern of P3 results for the second stimulus. In other words, the amplitude of the central-parietal P3 did not depend on the self-association of the stimulus that elicited the P3, but instead on the self-association of the preceding stimulus, regardless of whether this preceding stimulus was a label or a previously unknown face associated with one’s own person.

However, our P3 results to faces corroborate the findings of previous studies reporting enhanced P3 to the self-face in comparison with other (either familiar or unfamiliar) faces (Sui et al. 2006; Keyes et al. 2010; Tacikowski and Nowicka 2010; Cygan et al. 2021; Żochowska et al. 2021). Such an effect was also repeatedly found for one’s own face when compared to a close-other’s face, if—similarly to the present study—the close-other was freely selected by participants as their most significant person (Cygan et al. 2014; Kotlewska and Nowicka 2015; Cygan et al. 2021).

In the present study, one of the main differences between the processing of faces and shapes referred to the relation between the “self” and “close-other” conditions. While those two conditions differed in the case of faces, they did not differ in the case of shapes, as indicated by significant differences found both in the neural underpinnings and in RTs for faces and lack of such differences for shapes. The most obvious explanation of this dissociation refers to the familiarity of processed information. Specifically, the self-advantage found for the self-face vs. close-other’s face comparison was not observed if levels of familiarity of information referring to the self and the close-other were strictly equalized, as it was done for shapes. Thus, our P3 findings for the “self” and “close-other” conditions may be driven by the higher pre-experimental familiarity of one’s own face than the close-other’s face.

Moreover, this pattern of P3 findings may also be interpreted in reference to the attentional processing of information related to the self and close-other. Specifically, it has been proposed that the mechanisms boosting the prioritized processing of self-relevant information could be driven by automatic capture of attention and prioritized allocation of attention to self-related stimuli (review Humphreys and Sui 2016; Sui and Rotshtein 2019). Indeed, several studies have found that the self-face automatically captures attention (Tong and Nakayama 1999; Brédart et al. 2006; Alexopoulos et al. 2012; Wójcik et al. 2018; Wójcik et al. 2019; Alzueta et al. 2020), and numerous EEG studies have revealed greater P3 amplitude in response to one’s own face (Ninomiya et al. 1998; Sui et al. 2006; Tacikowski and Nowicka 2010; Kotlewska and Nowicka 2015; Żochowska et al. 2021; review: Knyazev 2013). The P3 is often associated with attentional processes (Polich 2007 but see Nieuwenhuis et al. 2005; Verleger et al. 2015), thus substantially enhanced P3 to the self-face, as reported in the current study, seems to reflect preferential engagement of attentional resources to one’s own face. In the case of shapes, similar P3 amplitudes for the “self” and “close-other” conditions may be linked to comparable attention allocation, i.e. the self-assigned shape did not benefit from such preferential allocation of attentional resources.

In general, interpretations of P3 findings referring to attentional processes are in line with the notion that P3 reflects stimulus processing only, i.e. with the view that P3 is a signature of a comprehensive evaluation of incoming stimuli (McCarthy and Donchin 1981; Duncan et al. 2009). This evaluation entails processes of allocation of perceptual and attentional resources to event encoding and categorization (Duncan-Johnson 1981; Donchin and Coles 1988), and P3 amplitude is assumed to reflect the amount of these resources or cognitive capacity involved in the stimulus evaluation (Isreal et al. 1980; Kok 2001). However, the current debate on the functional role of the P3 component is multifaceted and it refers to many different topics. Thus, other interpretations of P3 findings are also plausible. To start with, an alternative view is that P3 reflects some processes of stimulus–response (S-R) translation or integration, a bridging step between sensory encoding and response execution (Pritchard et al. 1999; Verleger et al. 2005). Following this general idea, it was proposed that P3 reflects (re)activation of well-established S-R links as in typical laboratory tasks, usually a few fixed S-R links or S-R schemas are established by instruction and practice (Verleger et al. 2014; Verleger et al. 2015). Such a link binds a stimulus-code with its corresponding response-code, leading to the automatic activation of the corresponding, already well-established, motor program, matching the presented visual stimulus (Hommel 2004), and this process is assumed to be reflected by P3 (Verleger et al. 2015). However, the design of our study was not intended to test the impact of S-R links on P3. Moreover, different patterns of P3 findings observed for familiar vs. unfamiliar shapes and faces did not provide any support for the S-R hypothesis.

Moreover, one may view P3 findings reported in the current study in the light of the locus coeruleus-noradrenergic (LC-NE) system activity. The pivotal role of the LC-NE system in regulating task engagement is well documented (Aston-Jones and Cohen 2005). Through its modulatory actions on information processing, the LC-NE system potentiates responses to the outcome of internal decision processes that involve motivationally significant events, thereby guiding behavioral action in the service of task demands and other goals (Aston-Jones and Cohen 2005). The modulatory effects of the LC-NE system may be measurable at the scalp as the P3 component. Thus, P3 is considered to be one of the psychophysiological markers of LC-NE activity (Murphy et al. 2011). Specifically, according to the LC-P3 hypothesis, the phasic activity of the LC and the resulting release of NE at axon terminals is critical in generating the P3 (Aston-Jones and Cohen 2005). It was also proposed that the P3 reflects the response of the LC-NE system to the outcome of internal decision-making processes and the consequent effects of noradrenergic potentiation of information processing (Nieuwenhuis et al. 2005).

So far, we viewed our P3 findings in the light of attentional mechanisms. However, this view may be complemented by the interpretation of P3 as reflecting processing of stimuli that are highly arousing in nature (Hu et al. 2011). These two interpretations—seeing the P3 amplitude as an index of attention or as an index of emotional arousal—are not mutually exclusive. According to Lang et al.’s (1997) model of motivated attention, emotional cues prompt motivational regulation and draw attentional resources. In fact, many behavioral (Armony and Dolan 2002) and electrophysiological (Cuthbert et al. 2000; Keil et al. 2002; Schupp et al. 2004; Briggs and Martin 2009; Foti et al. 2009; Hajcak et al. 2010; Franken et al. 2011) studies support this relationship between emotions and attention. Recent definitions of emotions emphasize their subjective character, i.e. emotions could be conceptualized as complex constellations of psychological and physiological states that reflect an organism’s appraisal of the meaning, relevance, and value of incoming stimuli (Dolan 2002). In this context, it is the motivational relevance of a particular stimulus to a particular person that determines the emotional vs. neutral evaluation. Our results for faces are in line with this interpretation: P3 findings may be attributed to the different emotional/motivational content of the self-face and other (close, unknown) faces, with the self-face being the most motivationally relevant.

As the P3 component reflects the cognitive evaluation of stimulus significance (Picton and Hillyard 1988; Mangun and Hillyard 1995; Bernat et al. 2001; Carretié et al. 2001), different patterns of P3 findings for faces and shapes (i.e. differences between the self-face and close-other’s face and the lack of differences between the self-assigned shape and the shape assigned to the close-other) may be due to the fact that new information associated with the self and the close-other evokes similar emotional responses and is characterized by similar levels of saliency, whereas the self-face is a more salient stimulus than the close-other’s face. Saliency of the self-face is often viewed as the primary driving factor of prioritized processing of that stimulus, and self-faces are among the most salient stimuli that we come across and process frequently (Devue and Brédart 2008; Apps et al. 2015; Wójcik et al. 2018; Wójcik et al. 2019). Self-relevant stimuli engage emotional processes and seeing one’s own face evokes a rather unique emotional response (Kircher et al. 2000). Such self-face advantage was observed even when the processing of one’s own face was directly compared to the processing of emotional (both happy and fearful) faces (Żochowska et al. 2021). Although in the current study the close-other’s face was chosen as an emotionally salient and overlearned non-self-face, the P3 and permutations tests differentiated these two faces. However, it was not the case for the self- and close-other assigned shape.

The limitations of the current study are as follows. Shapes were arbitrarily assigned to one’s own person and to the chosen close-other, and their processing was compared to unknown shapes. As those familiar conditions (the self and the close-other) are personally relevant, it is a matter of debate whether similar—or rather dissimilar—patterns of behavioral and electrophysiological findings would be observed for shapes assigned to famous people. Therefore, the inclusion of such an additional experimental condition would provide a more global view on the processing of newly acquired information referring to the self and others. Moreover, our study did not provide an answer to the question of whether the self-relevance of newly acquired information triggers the self-representation in the brain, similarly to highly familiar self-referential information (self-face). In order to adequately relate to this issue, some source analyses (i.e. dipole fitting, LORETA, CLARA) should be done. However, due to rather a low number of experimental trials, such analyses—in the case of our present study—would be not very reliable. Future EEG studies may investigate whether newly learned and long-term established self-related information are represented in the same (or overlapping) neural network in the brain.

In conclusion, P3 and permutation test results revealed a clear self-advantage in the case of faces, i.e. significant differences between the processing of the self-face and other faces (close-other’s, unknown). These findings may be viewed in the light of preferential attention allocation to highly familiar and well-established self-referential information. However, the processing of new information arbitrarily assigned to one’s own person and the close-other did not differ. We propose that this effect is mainly driven by similar attentional biases to self- and close-other assigned shapes.
